# Cognitive Training Deep Dive: The Impact of Child, Training Behavior and Environmental Factors within a Controlled Trial of Cogmed for Fragile X Syndrome

**DOI:** 10.3390/brainsci10100671

**Published:** 2020-09-25

**Authors:** Haleigh Scott, Danielle J. Harvey, Yueju Li, Yingratana A. McLennan, Cindy K. Johnston, Ryan Shickman, Joseph Piven, Julie B. Schweitzer, David Hessl

**Affiliations:** 1 MIND Institute, University of California Davis Medical Center, Sacramento, CA 95817, USA; hmoscott@ucdavis.edu (H.S.); yamclennan@ucdavis.edu (Y.A.M.); cindy.k.johnston@healthpartners.com (C.K.J.); rdshickman@ucdavis.edu (R.S.); jschweitzer@ucdavis.edu (J.B.S.); 2 Department of Psychiatry and Behavioral Sciences, University of California Davis School of Medicine, Sacramento, CA 95817, USA; 3 Division of Biostatistics, Department of Public Health Sciences, University of California Davis School of Medicine, Davis, CA 95616, USA; djharvey@ucdavis.edu (D.J.H.); yjlli@ucdavis.edu (Y.L.); 4 Department of Pediatrics, University of California Davis School of Medicine, Sacramento, CA 95817, USA; 5 Carolina Institute for Developmental Disabilities, University of North Carolina at Chapel Hill, Chapel Hill, NC 27599, USA; joe_piven@med.unc.edu; 6 Department of Psychiatry, University of North Carolina at Chapel Hill, Chapel Hill, NC 27514, USA

**Keywords:** FMRP, *FMR1* gene, intellectual disability, treatment, working memory, fragile X syndrome, cognitive training

## Abstract

Children with fragile X syndrome (FXS) exhibit deficits in a variety of cognitive processes within the executive function domain. As working memory (WM) is known to support a wide range of cognitive, learning and adaptive functions, WM computer-based training programs have the potential to benefit people with FXS and other forms of intellectual and developmental disability (IDD). However, research on the effectiveness of WM training has been mixed. The current study is a follow-up “deep dive” into the data collected during a randomized controlled trial of Cogmed (Stockholm, Sweden) WM training in children with FXS. Analyses characterized the training data, identified training quality metrics, and identified subgroups of participants with similar training patterns. Child, parent, home environment and training quality metrics were explored in relation to the clinical outcomes during the WM training intervention. Baseline cognitive level and training behavior metrics were linked to gains in WM performance-based assessments and also to reductions in inattention and other behaviors related to executive functioning during the intervention. The results also support a recommendation that future cognitive intervention trials with individuals with IDD such as FXS include additional screening of participants to determine not only baseline feasibility, but also capacity for training progress over a short period prior to inclusion and randomization. This practice may also better identify individuals with IDD who are more likely to benefit from cognitive training in clinical and educational settings.

## 1. Introduction

Fragile X syndrome (FXS) is a genetic condition associated with the full mutation of the fragile X mental retardation 1 (*FMR1*) gene. FXS occurs in an estimated 1 of every 4000 to 11,000 live births and is the most common inherited cause of intellectual disability [1]. Males tend to be more severely affected, with over 90% of males but only 30–50% of females with the full mutation having IQ scores in the intellectually disabled range (IQ < 70; [2]). Extensive research using both neuropsychological testing and functional magnetic resonance imaging (fMRI) studies has demonstrated the significant deficits in executive function (EF) associated with the condition. These deficits include problems with working memory (WM), inhibitory control, cognitive flexibility/perseveration and selective and divided attention [3,4,5,6]. While there has been extensive preclinical research and human clinical trials focused on potential disease-modifying pharmacological treatment, primarily focused on improving behavior, mood and anxiety, there has been limited research targeting cognitive function in FXS.

Cogmed is a computer-based WM training program that has been the subject of over 80 peer-reviewed publications. Randomized, double-blind, placebo-controlled studies have documented that Cogmed and other WM training procedures may improve WM and academic achievement, reduce symptoms in children with ADHD, increase auditory attention and WM in preschool children, and improve inattention in daily life [7,8,9,10]. While some research has supported these claims, the benefit of WM training programs remains controversial with other researchers arguing that improvements in training are not generalizable beyond the trained tasks [8]. However, as children with FXS have specifically demonstrated WM deficits, this cognitive intervention was seen to have the potential to ameliorate some of the EF problems in this population.

After a small noncontrolled trial demonstrated feasibility of Cogmed for children with FXS [11], we conducted a randomized controlled trial (RCT) of Cogmed training in 100 children and adolescents with FXS, and targeted WM, EF and behaviors associated with EF (attention, hyperactivity/impulsivity) as outcomes of interest [12]. Participants were randomized 1:1 to either the standard Cogmed program that adapts difficulty (memory span) according to performance (adaptive condition) or a control condition utilizing an identical version of Cogmed that does not adapt to performance, with each trial fixed at 2-span items (nonadaptive condition). Within adaptive and nonadaptive versions, participants received either Cogmed JM (generally for younger and/or lower functioning participants) or Cogmed RM (for older and/or higher functioning participants). Participants completed 5–6 weeks of training, totaling 20–25 (mean = 24.2) days at home supported by a parent training aide and supportive coaching by phone. At the group level, children with FXS in the adaptive condition were able to progress by gradually, though modestly, expanding their memory span while using the Cogmed games. However, considerable variability was observed across participants. Nonadaptive training was selected as the comparison condition, rather than a wait-list or treatment-as-usual condition, in order to control for potentially beneficial factors such as parent and coach input and attention to the child, expectation of treatment benefits and placebo response, and any general effects that may be associated with engaging in a computer task or game. The primary result of the trial showed that both the adaptive and the nonadaptive groups improved WM after the Cogmed training, but there was no difference in degree of improvement between groups. The intervention was feasible, and the full sample demonstrated significant improvements in WM and EF objective measures, as well as parent- and teacher-reported attention and EF. For full results, see [12]. One explanation for the gains in both groups and the lack of separation between adaptive and nonadaptive control conditions may be that a substantial number of children with FXS experience the nonadaptive condition as quite challenging and potentially beneficial. However, factors other than the training itself may have contributed to gains in both groups such as placebo or practice effects. Given the results of this study, with both groups experiencing improvement, we determined to conduct further analyses in an exploratory “deep dive” of this rich data set to better understand what factors were associated with improvements.

Cogmed is a well-researched cognitive training program. However, most studies have attempted to understand the significance of clinical outcomes by contrasting experimental groups [13,14]. Only a few studies have examined individual variability or any subcomponents of the training itself, and those have focused on the different types of games within the Cogmed program [15,16], with interest in identifying the specific aspects of WM that are targeted (e.g., verbal vs. visual spatial aspects). Only one study examined predictors of WM training in individuals with IDD [17]; this study showed that females and participants with an IDD but no additional diagnosis, on average, had more progress during training. Additional studies have evaluated other computerized cognitive training programs, such as Lumosity, but these have mainly evaluated patterns of performance by age [18,19]. To our knowledge, no published studies in any population have attempted to link training behavior parameters to outcomes, and no published study has examined potential effects of variation in the training environment on outcomes.

The current study used the detailed training behavior data from the FXS Cogmed RCT [12] and had three primary aims: (1) to characterize the training data, identify training quality metrics, and identify subgroups of participants with similar training patterns; (2) to identify predictors of training efficacy; and (3) to determine which child, training behavior, or home/environmental factors were associated with clinical outcomes during the Cogmed intervention.

## 2. Materials and Methods

### 2.1. Participants

Participants were 98 children with FXS that participated in the RCT of Cogmed; 2 of the original 100 participants were missing the detailed training data. Participants were between the ages of 8 and 18 years, with an average IQ of 64. They were 63% male, all with normal or corrected to normal vision and hearing and residing in various locations throughout the U.S. and Canada. For all relevant information on participants, see the original study 12].

### 2.2. Measures

The same primary and secondary outcomes from the original FXS Cogmed trial were used as the clinical outcomes of interest in the present study. These consisted of the Leiter-Revised [20] Spatial Memory subtest, the Stanford Binet 5 (SB-5; [21]) Block Span subtest; the Wechsler Intelligence Scale for Children, Fourth Edition (WISC-IV; [22]) Digit Span subtest; and the parent versions of the Conners Third Edition (Conners 3; [23]) and the Behavior Rating of Executive Function (BRIEF; [24]). The WM composite, comprised of the Spatial Memory and Block Span subtests, was the trial’s primary outcome measure. Teacher-reported behavior from the Conners and BRIEF and the Kiddie Test of Attentional Performance (KiTAP; [25]) were collected in the trial but not included in the present study due to insufficient sample size and limited power (only approximately 50% of the participants had teacher ratings).

In addition to the demographic, primary outcome, and secondary outcome measures reported in the original study, the following measures not previously reported were collected during the visits to further explore factors related to training success and clinical outcomes. The Home Observation for Measurement of Environment or HOME Inventory is an instrument designed to provide a systematic measurement of the family environment. The disability adapted Middle Childhood HOME Developmental Delay [26] was administered at the baseline assessment and consists of 59 questions generated from examiner observation and parent interview. The HOME Inventory covers the following domains: Responsivity, Encouragement of Maturity, Emotional Climate, Learning Materials and Opportunities, Enrichment, Family Companionship, Family Integration, and Physical Environment. The total score was used in the present study. The Symptom Checklist-90-Revised (SCL-90-R [27]) is a standardized self-report measure of psychological symptoms and was completed by the parent acting as a training aide as a self-report of parental mental health. Ninety questions are clustered into the following symptom dimensions: somatization, obsessive-compulsive, interpersonal sensitivity, depression, anxiety, hostility, phobic anxiety, paranoid ideation, and psychoticism. We examined the Global Severity and Depression scores for this study. The Parenting Stress Index-4 (PSI-4 [28]), also completed by the parent training aide, measures stress in the parent–child system based on parent’s perceptions of child characteristics, personal characteristics, and interactions between the child and parent. We focused on the parental distress and dysfunctional parent–child interaction scores.

To characterize training quality, the detailed Cogmed data from the 5–6 week training period for each participant were obtained from Cogmed. These data include summaries for each game at the level of each training day and trial-by-trial performance for each game played on each day.

### 2.3. Statistical Analyses

The first aim of the present study was focused on characterizing the training data and identifying clusters of participants with similar training behavior patterns. Four metrics were explored: (1) maximum trial difficulty achieved for each game, each day (adaptive group only, as the nonadaptive group had a fixed level); (2) response time on each trial for each game for each day of play; (3) standard deviation of response time for each game for each day of play (response time variability; see [29]); and (4) percentage of correct trials for each game for each day of play (accuracy). Trials for a particular game in which the response time was either negative (indicating a response before the end of the trial presentation) or greater than the 99th percentile (extreme delay in response; ranging from 3 seconds (s) to over 200 s) across all days the game was played by participants were considered invalid trials and removed from analyses, including computation of the standard deviation of response time. A sensitivity analysis was conducted removing trials with times greater than the 90th percentile and results were similar. Repeated-measures, random-effects models were used to assess general patterns over time and whether differences existed in those patterns between the adaptive and nonadaptive training groups. The standard deviation of response time was transformed using the natural logarithm prior to analysis to meet the assumptions of the models [30,31]. Time, in days since the first day of training, was used as the time scale for all models. Trial-level outcomes further included trial number as a factor. Models included random intercepts and slopes to account for variability in starting place and change over time not explained by the fixed effects.

Semiparametric mixture models were fit to the repeated measures at either the daily game level or the trial-by-trial level to identify clusters of training patterns for each outcome, separately for each game and for each training group [32]. Separate models were fit for the first three weeks of training (early training period) and the last three weeks of training (late training period), especially for trial-level data, due to software limitations. Bayesian information criterion (BIC) was used to select models and identify the number of subgroups present in the data; models with two, three, or four subgroups were considered. From the best models for each game, the likely subgroup for each participant for that game was also determined. Graphical illustrations for the subgroups suggested similar training patterns across games. Therefore, a single subgroup classification was assigned per participant as the most common identified subgroup across games.

For each of the four training metrics, trial difficulty level (adaptive group only), response time, standard deviation of response time, and accuracy training behavior/patterns were identified. Individuals that fell into one behavior group based on a training metric did not necessarily fall into the same group for another training metric, so individual participants were not classified into the same training behavior group across all training metrics. Instead, each training metric was evaluated separately to assess differences on child, parent/training aid and home characteristics using two sample t tests.

The last aim of this study was to determine which child, training, or home environment factors related to clinical outcomes (improvements in scores) reported in the trial. For demographic, parent, and home environment predictors, Time 2 (post-training) assessment was used as the outcome, with the Time 1 (pre-training) assessment as a covariate in an analysis of covariance (ANCOVA) model. Models further included total training time and treatment condition (nonadaptive vs. adaptive) as covariates. Separate models were run using each demographic, parent, and home environment factor as a predictor; interactions between the predictor and treatment condition were also considered. Similar models were fit to assess whether there were differences in clinical outcomes by training behavior groups. Secondary analyses considered training groups as predictors of clinical outcomes in each treatment condition separately.

## 3. Results

### 3.1. Training Metrics: Cogmed Adaptive and Nonadaptive Groups

Accuracy was lower in the adaptive group than the nonadaptive group in RM games but not significantly different in JM games at the first day of play, with the nonadaptive group increasing over time and the adaptive group remaining stable across training days (Table 1). Over most games, as expected, trial difficulty (adaptive group only) increased over time (Table 2). There were no differences between groups or changes with time in the standard deviation in response time (data not shown). For the trial-level data, the average response time decreased across the training days for most games (data not shown). Rate of decrease in response time did not differ by group (adaptive vs. nonadaptive).

### 3.2. Training Patterns

**Figure 1:**
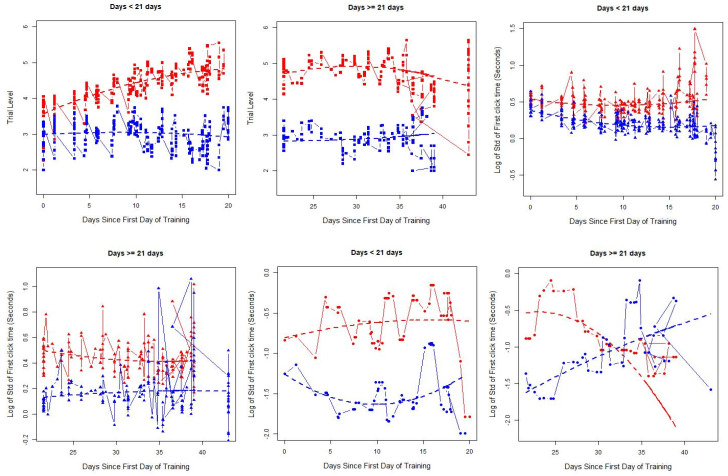
A panel of plots illustrating the underlying latent training groups identified for the different training metrics for Game 9 as exemplar in the adaptive group. Each plot contains the average at each time point (symbols) as well as the smoothed trajectory for the identified group (dashed line). Trial difficulty (solid squares), response time (solid triangles), and response time variability (solid circles) in the early (first 3 weeks) and late (after day 21) training periods are shown.

The optimal number of subgroups was identified using BIC based on data from the training period. Most games and outcomes suggested two subgroups, suggesting a two-cluster solution was appropriate. For examples of identified subgroups for a Game 9 in the adaptive group, see Figure 1; patterns were similar for other games as well as for the nonadaptive group. The first two plots, with trial difficulty on the *y* axis (within the adaptive group only), showed one group that had an essentially flat profile during both the early and late training periods (blue curve, no improvement) and another group that increased trial-level difficulty during the early training period and then stabilized or showed minimal decreases during the later training period (Figure 1). For response time (*y* axis), both groups had a slight decline in the early period and became more stable in the late training periods, but one subgroup (in blue) tended to have faster responses than the other subgroup (red). For response time variability, one group had smaller standard deviations (blue) than the other (red) in the early training period, where smaller standard deviation indicated more consistent response times across trials. In the later training period, the group with less variability initially (blue) increased over time, while the second group (red) showed a decline in response variability. One group (red) had much higher accuracy than the other group (blue) across the entire training period. Because patterns in the early and late periods generally reflected positive training behaviors (e.g., faster response time, lower variability in response time, better accuracy, and higher difficulty) and less positive training behaviors, we categorized individuals according to whether they remained in the “positive training behavior” group during both training periods or not for future analyses.

### 3.3. Demographics and Family Characteristics

For the trial difficulty metric, defined only for the adaptive group, those in the greater difficulty group (*n* = 14) had significantly higher IQ [mean of 74.3 (standard deviation (SD) = 20.9) vs. mean of 61.1 (SD = 15.2); *p* = 0.02] and higher mental age [mean of 9.6 (SD = 5.5) vs. mean of 6.5 (SD = 2.7); *p* = 0.01] than the rest of the adaptive group (*n* = 34). For the groups defined by the response time, including both those in the adaptive and nonadaptive groups, the faster response times group (*n* = 43) had parents with lower total stress raw scores from the PSI [mean of 80.6 (SD = 17.0) vs. mean of 91.1 (SD = 23.9); *p* = 0.02] and lower dysfunctional parent–child interaction scores from the PSI [mean of 25.9 (SD = 5.0) vs. mean of 29.8 (SD = 8.1); *p* = 0.006] than the remaining participants (*n* = 49). Finally, the low standard deviation response time group (*n* = 36) had higher mental age (mean = 8.1(SD = 3.8) vs. mean = 6.7 (SD = 0.34); *p* = 0.03) than remaining participants (*n* = 55). Those in the higher accuracy group (*n* = 70) had higher IQ [mean of 68.5 (SD = 17.9) vs. mean of 56.1 (SD = 8.0); *p* = 0.001] and mental age [mean of 7.7 (SD = 3.4) vs. mean of 6.0 (SD = 1.3); *p* = 0.02], but lower total stress raw scores from the PSI [mean of 83.4 (SD = 20.7) vs. mean of 96.2 (SD = 22.7); *p* = 0.01] than remaining participants (*n* = 24). No other parent or child variables were significantly related to outcome variables.

### 3.4. Predictors of Clinical Outcomes

For the WM composite (Leiter Spatial Span and Block Span) and Digit Span, there were no interactions between child, parent, or home environment variables and treatment condition, so results are presented for models containing no interaction. Higher baseline IQ was associated with greater gains in each of these outcome measures during the training period (*p* < 0.02). Higher mental age was also associated with greater gains on the outcomes (*p* < 0.01), except for Block Span which approached significance (*p* = 0.06). There were no significant predictors of change on the Parent BRIEF WM or Global Executive Composite (GEC) or the Connors scores. No other child, parent or home environment variables were significantly related to gains in outcome measures. See Table 3 for full results.

In the total sample (see Table 4), those with consistently faster response times had larger increases in Digit Span scores during the intervention (1 point greater), on average, than the remaining participants (β = 1.0; SE = 0.4; *p* = 0.02); this difference was significant after adjusting for IQ (β = 0.9; SE = 0.4; *p* = 0.03) but not after adjusting for mental age (β = 0.6; SE = 0.4; *p* = 0.1). Conners Inattention (β = −2.1; SE = 0.9; *p* = 0.03) scores decreased more in the faster responding group compared to other participants and remained significant after adjusting for IQ (raw: β = −2.1; SE = 0.9; *p* = 0.03; T: β = −3.9; SE = 1.9; *p* = 0.04) or mental age (raw: β = −2.1; SE = 1.0; *p* = 0.03; T: β = −3.8; SE = 1.9; *p* = 0.048). In the total sample, those in the low standard deviation in response time (those with consistently lower standard deviations) had WM gains that were 2.0 points higher, on average, than remaining participants (β = 2.0; SE = 1.0; *p* = 0.04), but not after accounting for IQ (β = 1.6; SE = 1.0; *p* = 0.1) or mental age (β = 1.4; SE = 1.0; *p* = 0.2). However, BRIEF GEC scores declined more in this lower standard deviation group (β = −6.7; SE = 3.0; *p* = 0.03) compared to the others, and remained significant after accounting for IQ (β = −6.8; SE = 3.2; *p* = 0.04) or mental age (β = −6.9; SE = 3.2; *p* = 0.04). There were no differences in gains in any of the clinical outcomes between the higher accuracy group and the remaining participants. Table 4 contains full results.

Follow-up identical analyses were conducted for adaptive and nonadaptive groups separately. These results revealed that the links between training behavior and outcomes were predominantly driven by significant associations in the adaptive, but not the nonadaptive group (see Table 5 and Table 6). For example, in the adaptive group only, the group defined by higher trial difficulty (those who showed progress in difficulty level over time with increasing span lengths) had WM composite score gains that were 3.9 points higher, on average, than the rest of the adaptive group (β = 3.9; SE = 1.6; *p* = 0.02; Table 6); this difference remained significant after accounting for IQ (3.5 points higher; SE = 1.6, *p* = 0.03) and mental age (3.1 points higher, SE = 1.6, *p* = 0.05).

## 4. Discussion

Whether computer-based cognitive training contributes to meaningful improvements in child functioning and quality of life remains a topic of considerable debate. Children with FXS are especially impacted by their cognitive deficits but have access to very few validated treatments, making the search for effective interventions to alleviate disability especially critical. Furthermore, a growing number of putative targeted pharmacological treatments for the disorder that might normalize brain function could be paired with structured cognitive therapy paradigms to examine whether these medications accelerate learning and cognitive growth. Our previously published study of the efficacy of WM training for children and adolescents with FXS, the first controlled trial of a cognitive intervention for the disorder, found that participants in both the adaptive and nonadaptive conditions demonstrated WM improvements on clinical assessment. Specifically, children in the nonadaptive condition, those who completed an identical intervention that did not adapt in difficulty according to performance, demonstrated gains and clinical improvement during the course of the trial that was similar to the adaptive group. This raised questions as to whether both interventions benefitted participants, or whether other factors may have explained improvements in the children. In the present study, we revealed numerous details regarding variability in the training behavior of participants, characteristics of their training environment and parent training aides, and the association of these variables with trial outcomes in order to provide greater insight into child individual differences in performance and outcomes, to clarify the factors contributing to gains in each intervention group, and to inform future studies.

The results of the present study demonstrate that baseline child characteristics as well as cognitive training behavior are associated with clinical changes during the intervention period. Training behavior metrics were linked not only to gains in WM performance-based assessments, but also to reductions in inattentive and other behaviors related to EF reported by caregivers. These patterns of association were stronger in the adaptive (experimental) training group. It should be emphasized that these analyses cannot confirm causal links between training behavior and clinical gains during training. However, the results do suggest that subgroups of children with FXS who can progress and expand memory capacity over time have better outcomes, perhaps better response to the intervention, than those who are unable to progress. Level of intelligence does not explain these effects fully, as several associations between training behavior and outcomes survived adjustment for baseline IQ. Therefore, future cognitive intervention trials with individuals with IDD should include additional screening of participants to determine not only baseline feasibility, but also capacity for training progress over a short period prior to inclusion and randomization. This practice would reduce the proportion of eligible participants but likely contribute to greater sensitivity to the efficacy of interventions and generalizability of results.

In terms of baseline child characteristics, only IQ and mental age were related to clinical outcomes with higher mental age and IQ being linked to greater gains in WM. This is similar to results reported by Söderqvist et al., who found that higher baseline ability was associated with greater working memory training gains in children with IDD [17]. As pointed out in that paper, the results run counter to literature in typically developing children which often reports that the individuals with lower baseline ability show the most improvement when provided with targeted training. It may be that children with greater cognitive impairment (those within the IDD range) need additional exposure to training (i.e., longer or more frequent) or they may need training in more than one cognitive domain to experience clinically meaningful benefits. Aside from IQ and mental age, no other child, parent training aide, or home environment variables were related to gains in the clinical outcomes of the study. To our knowledge, this is the first examination of potentially moderating home and parent factors on treatment outcomes in FXS. Although child outcomes were independent of these factors, it is worth noting that the majority of families had fairly high scores on the HOME inventory, suggesting that most home environments were positive and conducive for learning. Similarly, the majority of parent training aides had SCL-90-R and PSI scores in the average range. Therefore, children with FXS in more adverse home environments and with parents struggling with serious mental health issues or high levels of parenting stress were not adequately represented in this sample. Nevertheless, it may be useful for investigators and clinicians to be aware that these important parent and environmental metrics do not appear to have substantial impacts on child training outcomes within this study.

The analyses of training level data show that training behavior can reliably identify participant subgroups in several dimensions. Four metrics of training quality—difficulty, accuracy, response time and response time variability—were used to quantify training quality. Difficulty is a metric of advancement in training for the adaptive group, with some children progressing in difficulty over the course of trainings while others remaining relatively flat with no appreciable gains in performance. This stark difference was not appreciable in our prior group-level comparisons of the primary trial results, which suggested modest gains overall [12]. Children who displayed positive training behavior defined by advancement in difficulty had better clinical outcomes than those that did not, even after accounting for difference in baseline IQ. Therefore, it is unlikely that the clinical improvement seen in this subgroup is explained by their higher functional status. Thus, it is likely that the Cogmed training program is most appropriate and has greatest potential utility for individuals with FXS who are capable of increasing their WM span capacity. As noted previously, we utilized an inclusion criterion characterized by the ability to perform at least some 3-span items at baseline, reflecting an increased probability that the children had the potential to make gains beyond the nonadaptive level of 2 span. Given the results reported here, the demonstration of at least some short-term gains during an early exposure to the program may be the best indicator of potential benefit from sustained training during intervention. Similar results were found for the two other metrics of training quality, response time and variability in response time. These metrics are thought to be a measure of how attentive and engaged participants were in the games. Participants who were more attentive and engaged (faster response times and more consistency in response) were also more likely to show clinical gains after the completion of the training. These results are promising, as they show that quality of engagement with the training procedure may be a driving force behind clinical gains.

One of the important questions raised by the primary trial results is why the nonadaptive Cogmed group improved over the course of training. The findings of the present analysis do not establish consistent links between training behavior and outcomes in the nonadaptive group. One explanation for the lack of significant association may be decreased variability in training metrics in the nonadaptive group, as these participants were less challenged and had fixed trial difficulty. The nonadaptive group also had less variability in the primary outcome measures than the adaptive group (see Table 2 in [12]), perhaps making it more difficult to detect potentially meaningful correlations.

We considered the analyses reported here to be exploratory and as such, we did not adjust for multiple comparisons or tests. However, we note that all of the significant patterns of association between training behavior and outcomes (11/11 significant results) were in the expected direction (e.g., lower standard deviation of response time associated with gains in WM score or a reduction in BRIEF GEC score from baseline to end of intervention), suggesting a low likelihood of chance association. As we noted previously [12], we elected to use the nonadaptive Cogmed training as the control condition for this trial to determine efficacy. We did not include an additional comparison group—for example, treatment-as-usual or another contrast group—which is a limitation of the study. Cogmed is predominantly focused on WM as its target—although this is an important area in need of remediation for FXS patients, their deficits span a broad range of executive dysfunction. Interventions addressing multiple domains of function and perhaps in multiple contexts may be needed to produce robust effects that translate to improvements in quality of life. Although the original trial was powered to detect differences between treatment conditions, it was not powered to detect differences in associations between child/parent/home factors and clinical outcomes by treatment condition. Similarly, the training behavior groups detected in the analyses were relatively small in size, indicating limited power to assess differences in associations between these training behavior groups and clinical outcomes by treatment condition. Finally, the training behavior groups are data driven and may differ in other studies.

## 5. Conclusions

In summary, the present analysis afforded an opportunity to examine details of the cognitive training process and individual differences that are typically omitted from standard clinical trial reports. While the efficacy of Cogmed or other cognitive training programs for FXS and individuals with IDD remains an open question, the high-resolution training data we report allowed for identification of more- vs. less-responsive participants and further highlight possibilities to integrate cognitive training paradigms in treatment research for this population. Future cognitive training trial designs for FXS and IDDs should carefully consider the type of control condition utilized to examine training efficacy and perhaps more rigorous screening to ensure that participants are capable not only of performing cognitive tasks but also demonstrate capacity for making gains. The latter criterion may be analogous to determination that a drug of interest can engage its target in the population of interest before starting a trial. The results presented here lay the groundwork for a subsequent Cogmed trial that may require both a wait-list control group and a comparison condition that entails equivalent participant and caregiver contact and computer exposure but entails no cognitive training. Another potential design would be to capitalize on the demonstration that a subset of children with FXS expand their WM span over time, and pair the training with a targeted pharmacological intervention vs. placebo. In this scenario, the investigator may compare the slopes (degree of memory expansion or Cogmed indices of improvement) in those treated with medication vs. placebo to determine whether learning is enhanced by the drug.

## Figures and Tables

**Table 1:** Change over time in maximum number of trial sequences recalled correctly by game.

	Game	Version	Adaptive	Time	Adaptive X Time
9	rotating dots	RM	**−4.85 (1.05); −4.6 (34)**	**0.24 (0.03); 8.21 (34)**	**−0.22 (0.04); −5.5 (34)**
14	asteroids	RM	**−9.86 (1.26); −7.8 (34)**	0.05 (0.05); 1.06 (34)	−0.13 (0.07); −1.93 (34)
17	space whack	RM	**−2.78 (1.56); −1.78 (34)**	0.05 (0.03); 1.48 (33)	**−0.11 (0.05); −2.41 (33)**
29	visual data link	RM	**−4.94 (0.95); −5.2 (34)**	**0.21 (0.03); 7.94 (34)**	**−0.22 (0.04); −5.94 (34)**
30	data room	RM	**−5.50 (0.82); −6.7 (34)**	**0.23 (0.03); 8.59 (34)**	**−0.22 (0.04); −5.9 (34)**
31	input module	RM	**−5.69 (0.90); −6.33 (34)**	**0.22 (0.02); 8.73 (34)**	**−0.22 (0.04); −6.0 (34)**
32	input module w/lid	RM	**−5.2 (1.0); −5.38 (34)**	**0.21 (0.02); 8.67 (34)**	**−0.19 (0.03); −5.69 (34)**
33	rotating data link	RM	**−3.41 (1.38); −2.47 (34)**	**0.26 (0.03); 9.55 (34)**	**−0.25 (0.04); −6.43 (34)**
47	decoder	RM	**−4.08 (0.38); −10.84 (34)**	**0.68 (0.10); 7.04 (34)**	**−0.80 (0.14); −5.85 (34)**
53	sorter	RM	**−8.25 (1.09); −7.6 (34)**	**0.19 (0.04); 5.14 (34)**	**−0.23 (0.05); −4.55 (34)**
54	stabilizer	RM	**−3.11 (0.99); −3.14 (20)**	**0.24 (0.06); 4.23 (19)**	**−0.23 (0.08); −2.86 (19)**
58	3D cube	RM	**−8.91 (1.28); −6.97 (34)**	0.09 (0.05); 1.73 (34)	**−0.15 (0.07); −2.19 (34)**
85	animals	JM	0.27 (0.83); 0.33 (58)	**0.10 (0.03); 3.67(58)**	**−0.17 (0.04); −4.26 (58)**
86	bumper cars	JM	1.26 (0.81); 1.55 (58)	**0.12 (0.03); 4.57 (58)**	**−0.16 (0.04); −4.27 (58)**
87	ferris wheel	JM	1.03 (0.81); 1.27 (58)	**0.15 (0.03); 5.75 (58)**	**−0.17 (0.04); −4.63 (58)**
88	twister	JM	0.30 (0.84); 0.36 (58)	**0.11 (0.03); 3.91 (58)**	**−0.14 (0.04); −3.37 (58)**
89	rollercoaster	JM	0.61 (0.88); 0.69 (58)	**0.10 (0.03); 3.35 (58)**	**−0.15 (0.04); −3.51 (58)**
90	hotel	JM	0.29 (0.91); 0.32 (58)	**0.08 (0.03); 2.58 (58)**	**−0.17 (0.04); −4.11 (58)**
91	pool	JM	0.83 (0.98); 0.84 (58)	**0.09 (0.03); 3.41 (58)**	**−0.20 (0.04); −5.07 (58)**

β (SE); t(df) presented for each term in the model. The Adaptive column contains the average difference in the maximum number of trial sequences recalled correctly between the adaptive group and the nonadaptive group on the 1st day of training. The Time column contains the average change per day in the nonadaptive group. The Adaptive X Time column contains the average difference in change per day between the adaptive group and the nonadaptive group. Bolded values have *p* < 0.05.

**Table 2:** Change over time in maximum level achieved by game (adaptive group only).

	Game	Version	Time: β (SE); t(df) *	*p*-Value
9	rotating dots	RM	0.02 (0.005); 3.51 (17)	**0.003**
14	asteroids	RM	0.009 (0.009); 1.02 (17)	0.32
17	space whack	RM	0.05 (0.02); 2.45 (15)	**0.03**
29	visual data link	RM	0.02 (0.005); 2.97 (17)	**0.009**
30	data room	RM	0.02 (0.004); 4.99 (17)	**<0.001**
31	input module	RM	0.02 (0.004); 6.09 (17)	**<0.001**
32	input module w/lid	RM	0.01 (0.007); 2.23 (17)	**0.04**
33	rotating data link	RM	0.02 (0.008); 3.11 (17)	**0.006**
47	decoder	RM	0.003 (0.03); 0.11 (17)	0.91
53	sorter	RM	0.02 (0.006); 2.59 (17)	**0.02**
54	stabilizer	RM	0.03 (0.02); 1.76 (9)	0.11
58	3D cube	RM	0.02 (0.007); 2.38 (17)	**0.03**
85	animals	JM	0.01 (0.003); 3.81(29)	**<0.001**
86	bumper cars	JM	0.02 (0.004); 5.17 (29)	**<0.001**
87	ferris wheel	JM	0.02 (0.004); 4.74 (29)	**<0.001**
88	twister	JM	0.02 (0.003); 6.09 (29)	**<0.001**
89	rollercoaster	JM	0.02 (0.003); 4.96 (29)	**<0.001**
90	hotel	JM	0.01 (0.004); 3.23 (29)	**0.003**
91	pool	JM	0.01 (0.004); 3.56 (29)	**0.001**

* β (SE); t(df) presented for each term in the model. The Time column contains the average change per day. Bolded values have *p* < 0.05.

**Table 3:** Child, parent, or home environment as predictors of clinical outcomes.

	WM Composite	Digit Span	BRIEF GEC	BRIEF WM	Conners Hyperactivity	Conners Inattention
Parent 1 *: Less than College	0.34 (0.97); 0.35 (92)	−0.15 (0.43); −0.34 (91)	2.20 (3.21); 0.69 (81)	0.33 (0.61); 0.54 (89)	−0.50 (1.31); −0.39 (87)	0.52 (0.99); 0.53 (87)
Parent 2 *: Less than College	0.24 (1.00); 0.24 (87)	0.13 (0.45); 0.28 (86)	5.00 (3.17); 1.58 (78)	**1.33 (0.59); 2.25 (86)**	1.62 (1.29); 1.26 (85)	0.87 (0.97); 0.90 (85)
Household Income *: <$50K	2.11 (1.44); 1.46 (89)	0.65 (0.65); 1.01 (88)	3.88 (4.29); 0.90 (78)	0.86 (0.86); 1.01 (86)	−0.97 (1.98); −0.49 (84)	0.24 (1.44); 0.17 (84)
Household Income *: $50–100K	0.08 (1.08); 0.08 (89)	0.20 (0.49); 0.42 (88)	7.40 (3.41); 2.17 (78)	1.19 (0.66); 1.82 (86)	−0.78 (1.44); −0.54 (84)	0.44 (1.07); 0.41 (84)
Household Income *: Prefer Not to Say	1.88 (1.67); 1.13 (89)	0.69 (0.74); 0.92 (88)	6.26 (5.83); 1.07 (78)	0.89 (0.99); 0.90 (86)	0.48 (2.15); 0.22 (84)	2.34 (1.61); 1.46 (84)
Child Age	0.17 (0.15); 1.12 (93)	0.11 (0.07); 1.69 (92)	−0.46 (0.57); −0.81 (82)	−0.07 (0.10); −0.68 (90)	0.30 (0.22); 1.36 (88)	−0.01 (0.16); −0.08 (88)
Child IQ	**0.10 (0.03); 2.74 (91)**	**0.05 (0.02); 2.78 (91)**	−0.13 (0.11); −1.19 (80)	−0.01 (0.02); −0.65 (88)	−0.06 (0.04); −1.38 (87)	−0.06 (0.03); −1.92 (87)
Mental Age	**0.57 (0.19); 3.06 (90)**	**0.45 (0.08); 5.42 (90)**	−0.61 (0.63); −0.98 (79)	−0.01 (0.11); −0.11 (87)	−0.09 (0.25); −0.38 (86)	−0.22 (0.18); −1.28 (86)
Parent Total Stress	−0.007 (0.02); −0.33 (90)	−0.01 (0.009); −1.58 (89)	0.09 (0.09); 1.05 (81)	0.01 (0.01); 0.81 (88)	−0.02 (0.03); −0.64 (86)	0.01 (0.02); 0.52 (86)
Parent Distress	−0.008 (0.05); −0.15 (90)	−0.02 (0.02); −0.72 (89)	0.25 (0.18); 1.35 (81)	0.04 (0.03); 1.27 (88)	0.02 (0.07); 0.24 (86)	0.02 (0.05); 0.44 (86)
Dysfunctional Parent–Child Interaction	−0.002 (0.07); −0.03 (90)	−0.04 (0.03); −1.44 (89)	0.14 (0.26); 0.54 (81)	0.02 (0.04); 0.42 (88)	−0.10 (0.09); −1.09 (86)	0.02 (0.07); 0.24 (86)
SCL-90-R Global Severity	−0.18 (1.19); −0.15 (85)	0.56 (0.52); 1.08 (84)	2.55 (3.83); 0.67 (75)	0.10 (0.75); 0.14 (83)	−0.75 (1.57); −0.48 (81)	−0.87 (1.15); −0.76 (81)
SCL-90-R Depression	−0.19 (0.74); −0.26 (85)	0.22 (0.32); 0.69 (84)	1.43 (2.34); 0.61 (75)	0.24 (0.46); 0.51 (83)	−0.63 (0.97); −0.65 (81)	−0.67 (0.71); −0.95 (81)
HOME Total Score	−0.06 (0.06); −1.01 (77)	0.06 (0.03); 1.65 (76)	−0.13 (0.24); −0.53 (68)	−0.03 (0.05); −0.72 (76)	−0.08 (0.09); −0.89 (74)	−0.04 (0.07); −0.55 (74)

* Reference levels: Parent 1 education and Parent 2 education: college or higher; household income: >$100K. ANCOVA models included Time 1 outcome score, training type group, and total training time as independent variables predicting the Time 2 outcome score. Presented results are β(SE); t (df). Bolded values have *p* < 0.05. GEC—Global Executive Composite; WM—Working Memory; SCL-90-R—Symptom Checklist-90-Revised; HOME—Home Observation for Measurement of Environment; BRIEF—Behavior Rating of Executive Function.

**Table 4:** Training pattern groups as predictors of clinical outcomes (full sample).

	WM Composite	Digit Span	BRIEF GEC	BRIEF WM	Conners Hyperactivity	Conners Inattention
Response time	1.81 (0.97); 1.87 (88)	**§ 0.99 (0.42); 2.34 (87**)	−4.14 (3.11); −1.33 (79)	−0.01 (0.60); −0.02 (87)	−0.14 (1.33); −0.10 (84)	**§ −2.12 (0.94); −2.26 (84)**
Std dev response time	**2.04 (0.96); 2.12 (88)**	0.86 (0.46); 1.88 (87)	**−6.66 (3.01); −2.21 (79)**	−0.61 (0.60); −1.01 (87)	−1.42 (1.32); −1.07 (84)	−0.04 (0.98); −0.05 (84)
Accuracy	1.14 (1.16); 0.98 (89)	0.13 (0.53); 0.24 (88)	−3.56 (3.59); −0.99 (79)	−0.44 (0.70); −0.62 (87)	−1.90 (1.49); −1.28 (85)	−1.64 (1.10); −1.49 (85)

ANCOVA models included Time 1 outcome score, training type group (except for trial difficulty), and total training time as independent variables predicting the Time 2 outcome score. Presented results are β(SE); t (df), corresponding to the “positive training behavior” group compared to the “not positive training behavior group”. Bolded values have *p* < 0.05. § significant after adjustment for FSIQ.

**Table 5:** Training pattern groups as predictors of clinical outcomes in the nonadaptive group only.

	WM Composite	Digit Span	BRIEF GEC	BRIEF WM	Conners Hyperactivity	Conners Inattention
Response time	1.38 (1.26); 1.10 (42)	0.75 (0.64); 1.17 (41)	−0.93 (5.51); −0.17 (38)	0.79 (0.94); 0.84 (40)	1.33 (2.59); 0.51 (37)	−1.13 (1.71); −0.66 (37)
Std dev response time	1.22 (1.26); 0.97 (42)	0.04 (0.68); 0.05 (41)	−9.42 (4.91); −1.92 (38)	−1.16 (0.91); −1.26 (40)	−2.01 (2.21); −0.91 (37)	0.23 (1.58); 0.14 (37)
Accuracy	1.99 (1.27); 1.56 (43)	0.26 (0.70); 0.37 (42)	−6.51 (4.97); −1.31 (38)	−0.95 (0.91); −1.05 (40)	−3.05 (2.14); −1.43 (38)	**§ −3.21 (1.39); −2.31 (38)**

ANCOVA models included Time 1 outcome score, training type group (except for trial difficulty), and total training time as independent variables predicting the Time 2 outcome score. Presented results are β(SE); t (df), corresponding to the “positive training behavior” group compared to the “not positive training behavior group”. Bolded values have *p* < 0.05. § significant after adjustment for FSIQ.

**Table 6:** Training pattern groups as predictors of clinical outcomes in the adaptive group only.

	WM Composite	Digit Span	BRIEF GEC	BRIEF WM	Conners Hyperactivity	Conners Inattention
Trial difficulty	**§ 3.92 (1.59); 2.47 (43)**	0.44 (0.66); 0.66 (43)	−3.01 (3.99); −0.76 (38)	0.58 (0.89); 0.65 (44)	0.57 (1.67); 0.34 (44)	0.29 (1.35); 0.22 (44)
Response time	2.45 (1.51); 1.62 (43)	**1.45 (0.56); 2.59 (43)**	**§ −8.29 (3.37); −2.46 (38)**	−0.78 (0.79); −0.99 (44)	−1.80 (1.46); −1.24 (44)	**§ −2.99 (1.13); −2.6 (44)**
Std dev response time	**3.21 (1.51); 2.13 (43)**	**§ 1.70 (0.60) 2.81 (43)**	−4.26 (3.82); −1.11 (38)	0.07 (0.85); 0.08 (44)	−0.46 (1.66); −0.28 (44)	0.16 (1.30); 0.13 (44)
Accuracy	−0.32 (2.27); −0.14 (43)	0.37 (0.87); 0.43 (43)	3.61 (5.46); 0.66 (38)	0.77 (1.17); 0.66 (44)	−0.38 (2.16); −0.18 (44)	1.51 (1.79); 0.84 (44)

ANCOVA models included Time 1 outcome score, training type group (except for trial difficulty), and total training time as independent variables predicting the Time 2 outcome score. Presented results are β(SE); t (df), corresponding to the “positive training behavior group” compared to the “not positive training behavior group”. Bolded values have *p* < 0.05. § significant after adjustment for FSIQ.
